# Case Report: GPi DBS for Non-parkinsonian Midline Tremor: A Normative Connectomic Comparison to a Failed Thalamic DBS

**DOI:** 10.3389/fnhum.2021.709552

**Published:** 2021-08-03

**Authors:** Takashi Morishita, Yuki Sakai, Takayasu Mishima, George Umemoto, Michael S. Okun, Saori C. Tanaka, Yoshio Tsuboi, Tooru Inoue

**Affiliations:** ^1^Department of Neurosurgery, Faculty of Medicine, Fukuoka University, Fukuoka, Japan; ^2^Brain Information Communication Research Laboratory Group, Advanced Telecommunications Research Institute International, Kyoto, Japan; ^3^Department of Neurology, Faculty of Medicine, Fukuoka University, Fukuoka, Japan; ^4^Swallowing Disorders Center, Fukuoka University Hospital, Fukuoka, Japan; ^5^Departments of Neurology and Neurosurgery, Norman Fixel Institute for Neurological Diseases, University of Florida, Gainesville, FL, United States

**Keywords:** tremor, deep brain stimulation, normative connectome, globus pallidus, case report, ventral intermediate nucleus, thalamus

## Abstract

**Introduction:** The clinical efficacy of deep brain stimulation (DBS) for midline tremor has been heterogenous. Here, we present an atypical case with facial and palatal tremor treated with DBS. We aimed to show the difference between the fibers affected by stimulation of the two targets [globus pallidus interna (GPi) and ventral intermediate (Vim) thalamic nucleus] using a normative connectome analysis.

**Case Report:** A 76-year-old woman with a 4-year history of severe facial and palatal tremor due to craniofacial dystonia. Following a failed bilateral Vim DBS, explantation of preexisting leads and implantation of bilateral GPi leads resulted in the resolution of tremor symptoms following a failed bilateral Vim DBS. We performed a normative connectome analysis using the volume of tissue activated (VTA) as a region of interest. The results revealed that the fiber tracts associated with VTA of GPi DBS had connections with the facial area of the motor cortex while the Vim DBS did not.

**Conclusion:** This case study suggests the possibility that GPi DBS may be considered for midline tremor, and that the normative connectome analysis may possibly offer clues as to the structures underpinning a positive response. We may refine targets for some of the more difficult to control symptoms such as the midline tremor in this case.

## Introduction

Deep brain stimulation (DBS) is an effective treatment modality for medication refractory tremor disorders. The most common DBS target for tremor has been the ventral intermediate (Vim) thalamic nucleus region. The clinical efficacy for midline tremor however, has had heterogenous outcomes (Moscovich et al., 2013). In this report, we present an atypical case with facial and palatal tremor which was addressed by bilateral globus pallidus interna (GPi) DBS following failure of bilateral Vim DBS. We show the difference between the fibers affected by the two procedures using a normative connectome analysis.

## Case Description

A 76-year-old woman with a 4-year history of severe facial tremor accompanied by minimal tremor in all four extremities. The phenomenology was similar in appearance to jaw tremor in Parkinson's disease (PD) patients, but the dopamine transporter scan (DAT) showed no abnormalities. A laryngoscopic evaluation also revealed a palatal tremor. These tremors were similarly irregular in amplitude and frequency (3–6Hz), and considered to be a series of symptoms of a movement disorder. She was, therefore, diagnosed with tremor associated with craniofacial dystonia by movement disorders trained specialists. The tremor was refractory to maximally tolerated dosages of anti-parkinsonian medications, a beta blocker, and a benzodiazepine, so a surgical intervention was indicated. Following a multidisciplinary evaluation she underwent DBS surgery.

Bilateral Vim thalamic nuclei were selected for the initial DBS targets to address the midline tremulous movements, and the trajectory was planned to include the medial area of the Vim based on somatotopy (Morishita et al., 2020). A large anterior commissure (AC)-posterior commissure (PC) insertion angle was selected as previously suggested in the literature for this type of case (Moscovich et al., 2013). The patient visited clinic for DBS programing once a month or two, and stimulation intensity was increased to the near threshold level of stimulation-induced side effects. However, DBS therapy was ineffective for more than 2 years although there were no serious adverse events. Since she was suffering from severe social embarrassment due to tremor, she elected to undergo a revision DBS surgery. Based on phenomenology of her dystonic tremor manifesting as similar to PD and in an attempt to address her dystonia, we planned to explant the preexisting Vim DBS leads, and implant new GPi DBS leads bilaterally through the preexisting burr holes. Following DBS surgery, she visited the clinic once a month for DBS programming. The positive clinical effect manifested 2 months after surgery as the facial and palatal tremor nearly resolved. The tremor was hardly recognizable at 1-year follow-up (Supplementary Video 1). The DBS electrode position and the programming of the device at last visit following each procedure have been summarized in Table 1.

**Table 1:** The electrode position and the stimulation parameters at last follow up.

	**Vim DBS** ^*^				**GPi DBS**	
	**Left**		**Right**		**Left**	**Right**
**Lead position (tip of the electrode)**						
X	9.8		9.3		19.5	20.9
Y	−3.9		−3.9		2.4	4.8
Z	−0.6		−0.2		−4.4	−5.4
AC-PC angle	69.9		65.5		74.7	78.5
Center-Line angle	32.5		32.0		19.9	14.0
**DBS programming**	**Program 1**	**Program 2**	**Program 1**	**Program 2**		
Active contacts	0+, 1−	2−, 3+	0+, 1−	2−, 3+	2−, Case+	2−, Case+
Amplitude (V)	2.8	3.1	2.7	3.0	3.8	3.8
PW	180	180	180	180	100	100
Frequency	100	100	100	100	130	130

* *Interleaving stimulation settings were applied. AC-PC, Anterior Commissure-Posterior Commissure; DBS, Deep Brain Stimulation; GPi, Globus Pallidus Interna; PW, Pulse Width; Vim, Ventral Intermediate (nucleus of the thalamus)*.

## Normative Connectome Analysis

**Figure 1:**
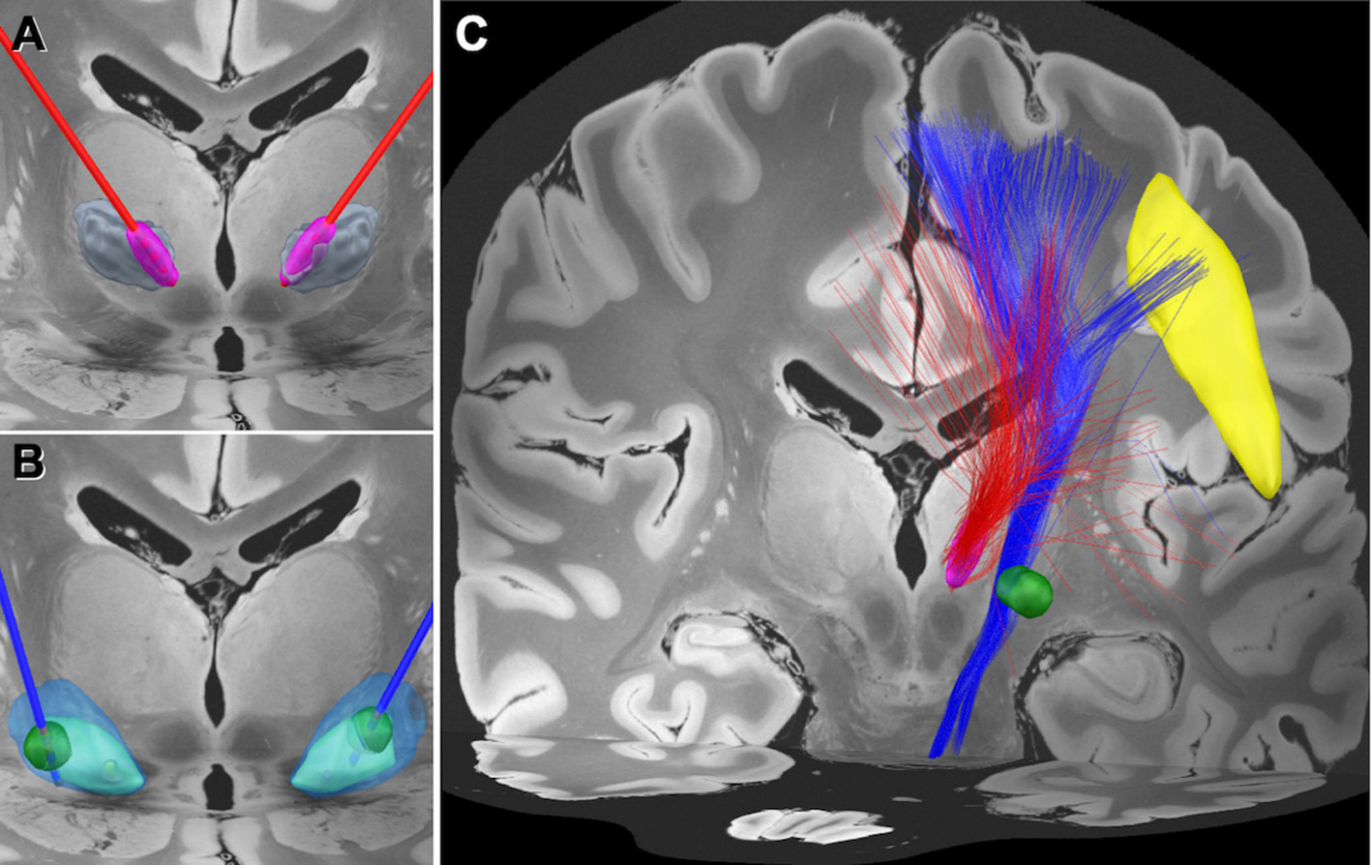
Electrode position and the normative connectome associated with each volume of tissue activated. **(A)** Position of the electrodes (red) and the volume of tissue activated (VTA) (magenta) in the medial area of the ventral intermediate (Vim) nucleus of the thalamus (gray). **(B)** Position of the electrodes (blue) and the VTA (green) in the globus pallidus interna (GPi) (light blue) and externa (GPe) (transparent blue). **(C)** Normative connectome associated with VTA of Vim stimulation (red tractography) and GPi stimulation (blue tractography). The normative connectome associated with GPi stimulation projects to the facial area of the motor cortex (yellow) and the brain stem. The facial area was defined by the A4hf region of the human brainnetome atlas (Fan et al., 2016).

Based on the clinical outcomes, we hypothesized that there would be a difference in the modulated networks between the Vim and GPi DBS. We, therefore, performed a normalized connectome analysis using the volume of tissue activated (VTA) as a region of interest (Morishita et al., 2021). Considering the limited data volume, the right VTAs were non-linearly mirrored and merged with the left VTAs on an assumption that there is no significant difference in functional localization between the left and right hemispheres. The VTA calculation was performed using Lead-DBS (Horn et al., 2017), and the population-averaged atlas of the macroscale human structural connectome derived from the diffusion-weighted imaging data (Human Connectome Project: https://www.humanconnectome.org/) (Yeh et al., 2018), was used for normative connectome analysis. The results revealed that the fiber tracts associated with VTA of GPi DBS had connections with the facial area of the motor cortex as defined by the A4hf area of the human brainnetome atlas (Fan et al., 2016). While the Vim DBS did not have connections, the tractography associated with the VTA for the GPi DBS may also have connections to the corticobulbar tract (Figure 1). We evaluated the number of structural fibers that passed through each brain region defined by Harvard-Oxford cortical/subcortical atlases (Makris et al., 2006) combined with the cerebellum from AAL atlas (Tzourio-Mazoyer et al., 2002). All small parcels of the cerebellum defined in AAL were integrated into one binarized parcel in a similar manner to our previous report (Morishita et al., 2021). The depicted fiber tracts showed that the VTA for GPi DBS had more connective fibers with the precentral gyrus and brain stem than the VTA of Vim DBS. The presented study participant provided informed consent, and this study design was approved by our institutional review board (IRB) (IRB approval number: U21-01-003).

## Discussion

This case study suggests the possibility that in some cases GPi DBS may be considered for midline tremor (Patel et al., 2018), and that the normative connectome analysis may possibly offer clues as to the structures underpinning a positive response. This result also suggests that the GPi DBS modulates the corticobulbar tract that connects these regions. To the best of our knowledge, this is the first report showing the differences in the potential mechanisms of action between Vim and GPi DBS in the same tremor patient. We hypothesize that the failure of the first procedure was due to the lead positioning when compared to the overlap of fiber tracts associated with GPi DBS. It is also possible that the DBS electrodes were not positioned in the appropriate area of the Vim nucleus as there have been a variety of thalamic DBS targets inclusive of ventralis oralis and ventralis caudalis nuclei (Morishita et al., 2010).

There have been arguments regarding DBS targets for dystonic tremor including Vim and GPi (Fasano et al., 2014). A recent study reported that Vim DBS may fail to show sustained suppression of dystonic tremor (Cury et al., 2017). Even though GPi DBS outcomes have been also heterogenous (Fasano et al., 2014), GPi DBS may be currently indicated as the first-line DBS target in cases with midline dystonic tremor. It should be also noted that cerebellar DBS might be an option for patients who are refractory to the conventional DBS treatment (Horisawa et al., 2021). Further clinical studies are warranted to identify the most effective target for dystonic tremor.

## Limitations

Even though our case study showed the beneficial effect of GPi DBS underpinned by the normative connectome analysis, there are important limitations. This is a retrospective study using the clinical data, and the stimulation parameters were not optimized based on the simulation by the connectome analysis. The recent studies showed that the different functional connectivities are affected between essential tremor and dystonic tremor (Tsuboi et al., 2021), but normative connectome analysis does not take the patient-specific network abnormalities into account. The normative connectome data were also descriptive and not quantified. Therefore, a larger sample size is necessary to confirm our findings with quantitative analysis. The normative connectome analysis, however, provided us with useful information to guide future studies. Though this is a case study, if we continue to use this type of 3-D approach, the hope is that long-term we can refine targets for some of the more difficult to control symptoms such as the midline tremor in this case.

## Data Availability Statement

The datasets presented in this article are not readily available because the personal data including the imaging data obtained from the study subjects will not be distributed openly to protect the patients' privacy. Requests to access the datasets should be directed to https://www.lead-dbs.org/.

## Ethics Statement

The studies involving human participants were reviewed and approved by Fukuoka University-Medical Ethics Review Board. The patients/participants provided their written informed consent to participate in this study. Written informed consent was obtained from the individual(s) for the publication of any potentially identifiable images or data included in this article.

## Author Contributions

TMo and YS designed the study and co-wrote the manuscript. TMo, TMi, and GU collected the clinical data. YS and ST contributed to the image analysis. MO and YT confirmed the diagnosis of the case. TI supervised this study. All authors critically reviewed the manuscript and approved the submitted version.

## Conflict of Interest

The authors declare that the research was conducted in the absence of any commercial or financial relationships that could be construed as a potential conflict of interest.

## Publisher's Note

All claims expressed in this article are solely those of the authors and do not necessarily represent those of their affiliated organizations, or those of the publisher, the editors and the reviewers. Any product that may be evaluated in this article, or claim that may be made by its manufacturer, is not guaranteed or endorsed by the publisher.

## Supplementary Material

Supplementary Video 1 On this clip, pre- and post-operative symptoms are presented on the left and right, respectively. Prior to surgical intervention (2 months prior to surgery), the tremulous movements are recognized in the chin, eyelid, and the palate. All of these symptoms were improved following GPi DBS (1 year following surgery).
